# Discoloration Mechanisms of Natural Rubber and Its Control

**DOI:** 10.3390/polym14040764

**Published:** 2022-02-16

**Authors:** Mingzhe Lv, Lei Fang, Heping Yu, Porntip Rojruthai, Jitladda Sakdapipanich

**Affiliations:** 1 Department of Chemistry and Center of Excellence for Innovation in Chemistry, Faculty of Science, Salaya Campus, Mahidol University, Phutthamonthon, Nakhon Pathom 73170, Thailand; lvmz12@163.com; 2 Institute of Agricultural Product Processing Research, Chinese Academy of Tropical Agricultural Sciences, Zhanjiang 524001, China; fanglei789-2002@163.com (L.F.); lhc2012168@163.com (H.Y.); 3 Division of Chemical Industrial Process and Environment, Faculty of Science, Energy and Environment, King Mongkut’s University of Technology North Bangkok, Rayong 21120, Thailand; porntip.r@sciee.kmutnb.ac.th

**Keywords:** natural rubber, discoloration, color substances, enzymatic browning, polyphenol oxidase, Maillard reaction

## Abstract

Color is an important indicator for evaluating the quality of natural rubber (NR). Light-colored standard rubbers are widely used in high-grade products and have high economic value. This paper first introduces the history and test standards of the standard light-colored rubber. The origin of color deepening in NR processing, color substances, and its biosynthetic pathway are reviewed. Then, the discoloration mechanism of NR is studied from the perspectives of enzymatic browning (caused by polyphenol oxidase and polyphenols) and non-enzymatic browning (including Maillard reaction and lipid oxidation). Finally, the strategies to control the discoloration of NR will be described.

## 1. Introduction

Natural rubber (NR) latex obtained by tapping the bark of the rubber tree contains rubber hydrocarbon and many non-rubber substances, including other organic substances such as carbohydrates, lipids, proteins, amines, carotenoids, phenolic substances, and also some inorganic constituents.

After naturally occurring coagulation or adding some coagulants such as acid into the NR latex, the solid NR is on occasion distinctly yellow in color; some may turn black. Color is one of the important indicators to evaluate the quality of NR. This characteristic is a critical key to determining the standards and trading prices. Certain types of NR, such as crepe rubber, are graded visually based on the lightness in the color of the dry rubber. Color is also an important sensory index of NR products, which directly affects human visual and psychological perception of the products and their application in some fields. Light-colored and colored filled products require pale rubber, light or extra-light graded rubber as feedstock, which is produced by bleaching during the production process.

Light-colored standard rubbers such as XL and L grades are produced from field NR latex with well-controlled technical specifications, including the restriction of the color index. It is an excellent rubber for manufacturing light-colored products, especially medical, health care, food, and high-grade daily uses.

## 2. History of Standard Light-Colored Rubber

The Standard Malaysian Rubber (SMR), developed in 1965 by the Rubber Research Institute of Malaysia (RRIM), was the pioneer of the process classification of NR. In 1970, Malaysia began to revise the SMR first, adding two grades of light-colored rubber, i.e., SMR5L (a light-colored subgrade of SMR5) and SMREQ (an extra-clean and light-colored rubber). From 1977–1978, with the participation of producers, merchants, and users, the technical specifications of SMR were revised for a second time. The revised standard limits impurities, ash, and volatile contents in light-colored rubber and changed to SMRL. By the 1980s, RRIM had developed nine varieties and 11 specifications, of which SMRL accounted for about 7–8% of the total SMR production. Influenced by Malaysia, other major rubber-producing countries worldwide are developing their standard rubbers, increasing the production of standard light-colored rubber to meet the needs of users, and gradually realizing quality standardization. For example, SIR5L from Indonesia accounts for about 6% of the total production of Standard Indonesian Rubber (SIR), and STR5L of Thailand accounts for about 2–3% of the total production of Standard Thai Rubber (STR). The acronym “L” stands for light-colored from NR latex as raw material for production [1].

According to the proposal and methods of SMR, the International Organization for Standardization (ISO) approved ISO 4660-1977 as the international standard for the determination of the color index of standard light-colored rubber. In 1978, ISO 2000-1977 was revised to add light-colored No.5 rubber and its specifications, and ISO 2000-1978 was published. Since then, standard light-colored rubber has become one of the advanced rubber varieties for international trade [2].

## 3. Test Methods for the Color of Natural Rubber

Light-colored standard rubber is the superior grade of standard rubber with color specifications, i.e., a light color is the most important characteristic of light-colored rubber. At present, the standard of most countries specifies the color of standard light-colored rubber and its test methods, which are usually expressed by the Lovibond color index [3,4]. The color index for NR is the scale value of the color obtained by comparing the sample of specified thickness and shape with the standard color glass under diffused light. The CIE L* a* b* color space introduced by the International Illumination Commission (CIE 1976) has become the universal color measurement standard globally adopted by all countries. L* defines the lightness value, a* and b* denote the red/green and yellow/blue values, respectively. It is suitable for all light source color or object color expressions and for accurate measurement [5], so it can measure the color of NR. The causes of NR color formation are complicated, in general relating to rubber clones, processing technology, impurity content, storage period, and other factors. The value change trend of lightness L* and color difference ΔE*ab corresponds to the color depth of the sample. Correlation study showed that color parameters of L*, b* (positive value in NR, yellowness), ΔE*ab (color difference), C* (chroma), and ΔH* (metric hue difference) value are negatively correlated with rubber impurities and ash content, negatively correlated with volatile content, and weakly positively correlated with nitrogen content; a* is a weak negative correlation with impurity content [6]. This is due to the origin of the color of NR.

## 4. Color Source in Natural Rubber

If we consider the whole production process of NR, in addition to the processing technology and the increased impurity and ash contents caused by the human operation, the color substance of NR itself and the discoloration during processing and storage are the main reasons for the final yellow-brown appearance.

### 4.1. Processing Technology

Certain preservatives will darken the color of NR. For example, when formaldehyde is used as a preservative, it will blacken the rubber even when the dosage is less than 0.05%. The effect of ammonia-stabilized latex was previously shown to affect the surface composition of rubber particles, which will cause the changes in rubber color depends [7,8]. When the amount is high and the storage time is long, the rubber color becomes dark. In addition, early preservation, improper processing, or other external factors may lead to latex corruption. Premature solidification makes it difficult to remove the impurities contained in NR, resulting in a deepening of the color of the rubber.

### 4.2. Impurities and Ash

The ash and impurity contents of NR are the inorganic salts (potassium, sodium, calcium, magnesium, aluminum, and phosphoric acid or sulfate of copper, manganese, iron, and other metal elements) and exogenous impurities (mainly sediment and rust, etc.) existing in the rubber. The content of inorganic salts in rubber depends on cultivation factors, and foreign impurities are often the main cause of high ash content. These substances will develop the color of the rubber. The artificially introduced impurities and ash lead to the darkening of the color of NR. The resulting accelerated aging of metal ions further exacerbates the change. The long-storage NR starts auto-degradation, and the appearance of the color deepened, even when it was kept under mild conditions in the storage room, i.e., no light exposure [9].

On latex application to textiles, pink-brown stains developed after some months of production. In the presence of light, ferric hydroxide forms with the rubber hydrocarbon, with an oxidation product of the rubber hydrocarbon, or with some substance (acetone- and water-insoluble) intimately associated with it, a pink-brown colored compound [10]. Although this investigation is incomplete, it illustrates that light was a likely contributory factor in rubber discoloration, although this fact did not entirely rule out the iron.

### 4.3. Aging

Under the physical action of high temperature, light, radiation, or mechanical force, NR causes the main chain or side groups of rubber macromolecules to break, which is the aging of natural rubber. Aging brings changes in appearance and deterioration of properties to NR. The various types of the aging breakdown of rubber were described in 1945 [11], including surface changes such as discoloration and frosting and changes throughout the rubber such as loss of strength, hardening, and softening. The possible cause of surface change is that the sample is exposed to light Attack by oxygen or possible vulcanization catalyzed by light. Several of the effects cited above occur simultaneously and may contribute jointly to the final result. Therefore, we can only generally think that NR discoloration is closely related to aging in the natural environment.

It is well-known that rubbers containing unsaturated structures are easily excited by ultraviolet (UV) light to generate free radicals [12]. UV light at a wavelength of 300 nm can effectively excite the active hydrogen of NR, cause it to oxidize, and generate oxygen-containing groups [13]; it then becomes sticky or tacky. However, there is no evidence of a direct cause-and-effect relationship between UV light and discoloration.

### 4.4. Container of Latex

The container of latex also brings about a color change. Rhodes and Sekar [14] have discussed the causes of discoloration in preserved latex fully and considered that the iron drum for shipment is a predisposing factor in the discoloration of NR latex. Discoloration in the preserved latex is attributed to iron contamination and the presence of hydrogen sulfide or soluble iron-reactant sulfides.

However, users were reluctant to replace steel drums with non-metal containers due to cost considerations. They have been used ever since, but there were some efforts to reduce to the minimum effects from the iron drum by the internal application of some protective medium [15].

### 4.5. Color Substances in Natural Rubber

It is widely believed that NR gives naturally occurring color in the biosynthesis process. Therefore, the characterization of color substances presented in NR is useful for developing a certain methodology to eliminate them completely or partly from NR.

#### 4.5.1. Composition and Biosynthesis of Color Substances

Total carotenoids from the neutral lipids have been identified as the primary pigments that result in a darker processed latex color [16]. Sakdapipanich et al. [17]. have tried to purify and characterize the color substances extracted from various fractions of *Hevea* rubber latex by certain methods using high-resolution characterization techniques. It was found that the content of color substances extracted was highest for the bottom fraction (BF) (0.5326% *w*/*v* of fresh latex, FL), followed by rubber cream (0.1996% *w*/*v* of FL), and Frey Wyssling (FW) particles (0.0629% *w*/*v* of fresh latex, FL), while the contents of the extracts from FL and STR20 were 0.8326% *w*/*v* of FL and 1.9900% *w*/*w* of dry rubber, respectively.

According to the FTIR, ^1^H- and ^13^C-NMR analyses, the color substances extracted from NR were composed of carotenoids, tocotrienol esters, fatty alcohol esters, tocotrienols, unsaturated fatty acids, fatty alcohols, diglyceride, and monoglyceride with different amounts and shades of color. This indicates that the color substances in NR are not only carotenoids as recognized early on, but they are composed of various kinds of substances, as mentioned above. It was also reported that polyphenols, proteins, and carotenoids are the coloring constituents in NR latex [17]. The concentration of polyphenols, proteins, and carotenoids in the NR latex were 2 × 10^−2^, 1, and 3 × 10^−5^ (*w*/*w*), respectively, and their contribution to the total absorptivity in the yellow-brown region were 2.3, 0.1, and 0.01%, respectively [18]. After processing the NR latex into the dry rubber, the residual color substances continue to be oxidized by heat to generate a variety of yellow oxides; the value of the color index with the clones and processing parameters are very different.

#### 4.5.2. Biosynthesis of Color Substances

**Figure 1:**
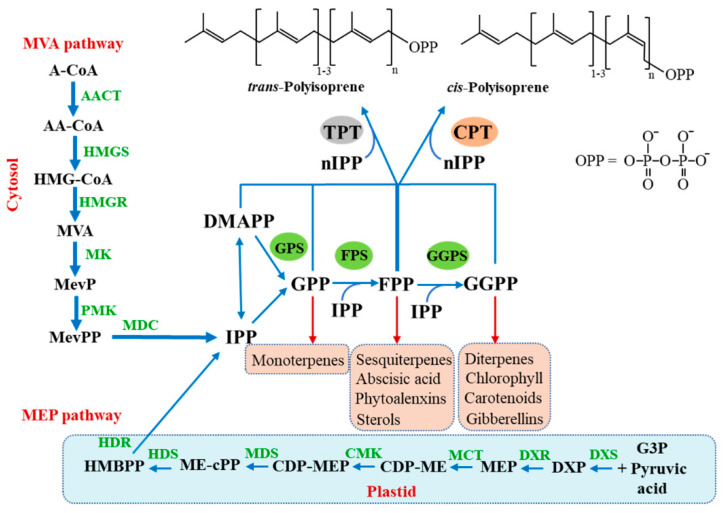
Biosynthesis of NR via the cytosolic mevalonate (MVA) pathway and the 2–C-methyl–d–erythritol 4–phosphate (MEP) pathway. Adapted from [19].

The pathway of rubber biosynthesis can be concerned with two components, isoprenyl diphosphate (IPP) and its isomer dimethylallyl diphosphate (DMAPP), synthesized from central intermediates. IPP and DMAPP are synthesized by two completely different pathways. Only the mevalonate (MVA) pathway has been considered responsible for IPP and DMAPP biosynthesis. Nevertheless, the methylerythritol phosphate (MEP) pathway was detected and deciphered. Plants possess both pathways; the MEP pathway is expressed in the plastids, and the MVA pathway is expressed in the cytoplasm. After the process above, the rubber molecule can be polymerized by subsequent additions of IPP to DMAPP via geranyl diphosphatase (GPP), farnesyl diphosphatase (FPP), and geranylgeranyl diphosphatase (GGPP) catalyzed by prenyltransferases. However, IPP and DMAPP can be proceeded on another pathway via GPP, FPP, and GGPP, as exhibited in Figure 1 [19].

The allylic-PP could provide various non-rubber compounds, including carotenoids, previously mentioned as the origin of the color substances. Nevertheless, the exact mechanisms for each pathway have not been recognized. According to the previous results, considering the microstructure of tocotrienols and their derivatives, which were also reported as color substances, they were classified as isoprenoids compounds due to the isoprenoid unit within their structures. Therefore, it can be proposed that these compounds were possibly derived from the NR biosynthesis pathway, as discussed previously.

**Figure 2:**
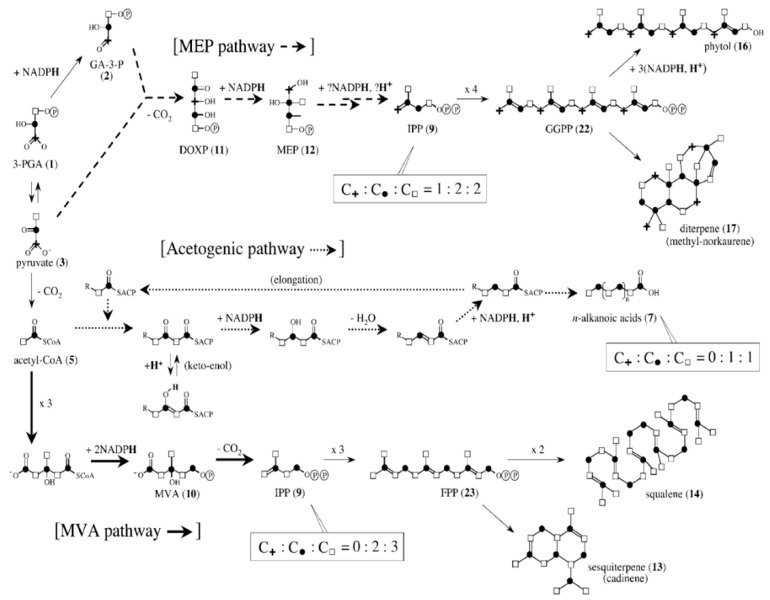
The relationship between carbon and hydrogen sources for positions in the specific lipid biomolecules associated with acetogenic, MVA, and MEP pathways. Adapted from [20].

In higher plants, typical lipids biomolecules were related to three different biosynthesis pathways, as shown in Figure 2. The *n*-alkanoic acids were biosynthesized in the acetogenic pathway with an acetyl-CoA biosynthetic precursor. They include n-alkanes, n-alkanols, and n-alkanoic acids. The *n*-alkanes and n-alkanols were biosynthesized from n-alkanoic acids by enzymatic decarboxylation and reduction, respectively. Hence, fatty acids, fatty alcohols, and derivatives were probably further generated from this step by such mechanisms. In contrast, all isoprenoid lipids were constructed from IPP, which was generated as mentioned above [20].

## 5. Discoloration Mechanism of Natural Rubber

Plant latex is a milky-like fluid (sap) stored in plant specialized cells-laticifers. It contains a mixture of phytochemicals, proteins, and enzymes, such as alkaloids, phenolics, terpenoids, defense proteins, proteases, and chitinases. Most of these latex ingredients possess pharmacologic activity. The NR latex mainly derived from *Hevea brasiliensis* has all the common properties of latex.

When food or other substances are processed or stored in a humid and hot environment for a long time, the amino compounds, such as proteins, amino acids, aldehydes, and ketones, meet with reducing sugars and produce brown polymers through a series of reactions called a browning reaction. According to the mechanism, the major reactions leading to browning are enzymatic phenol oxidation and so-called non-enzymatic browning.

### 5.1. Enzymatic Browning

**Figure 3:**
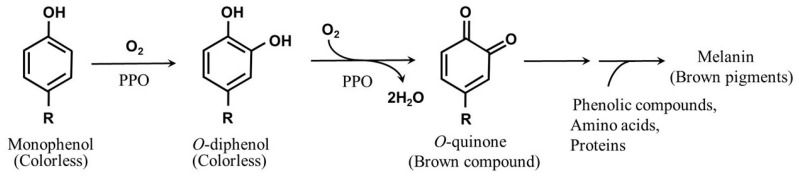
Brown pigment (melanin) formation from phenolic compounds.

NR causes discoloration during processing, similar to that in food processing. It is mainly due to the phenols and aminophenols in the latex combining with oxygen to form *o*-quinones under the catalysis of polyphenol oxidase (PPO) [21]. The substrates (mainly polyphenols) that participate in enzymatic browning are present in plastids, whereas the enzymes are located in the cytoplasm. PPOs interact with the substrates flowing out of the plastid during processing. Phenolic substances in plants are oxidized to quinone under the catalysis of phenolase and peroxidase, and the quinone undergoes a non-enzymatic reaction (to be discussed later) to produce brown pigments, such as melanins [22]. The reaction mechanism of enzymatic browning is shown in Figure 3. The extensive difference in the hue and intensity of color pigments relies on the source of polyphenols and environmental causes of the oxidation process during the browning process [23].

Afterward, non-enzymatic reaction with molecular oxygen gives rise to auxiliary reactions of formation of complex products, such as indole-5,6-quinone from tyrosine. Then, *o*-benzoquinones covalently react with another polyphenol to provide intensive-colored compounds ranging from red, yellow, green, and blue, to black. *O*-benzoquinones, upon their reaction with thiol compounds and aromatic amines and those in proteins, provide a great range of products, consisting of high-molecular-weight protein polymers.

The *o*-quinone, which leads to phenolic discoloration, is blocked by glutathione (γ-glutamyl cysteinyl glycine, GSH), and it is reduced by *o*-quinone back to catechol. If GSH is not present, *o*-quinone can react with other phenolic compounds or amino acids (in protein) or self-polymerize into melanin-like brown pigment [24]. With an increase in L-cysteine concentration, there is a corresponding decrease in the rate of browning reaction and PPO activity. The proposed mechanism is confirmed by the FTIR spectrum of acetone extract of the rubber sample.

Researchers have gradually deepened their understanding of the discoloration mechanism of NR. In the beginning, they did not know what role PPO or polyphenol substances played but found in their research that the discoloration of NR was related to the content of polyphenol substances or polyphenol oxides.

#### 5.1.1. Polyphenol Oxidase (PPO)

Total carotenoids from the neutral lipids have been identified as the primary pigments that result in a darker processed latex color [13]. Sakdapipanich et al. [14] have tried to purify and characterize the color substances extracted from various fractions of *Hevea* rubber latex by certain methods, using high-resolution characterization techniques. It was found that the content of color substances extracted from FL, rubber cream, the BF, FW particles, and STR 20 was different [17]. 

PPO is a copper-binding metalloproteinase widely distributed in plants, animals, fungi, and other cells, which plays a role in plant defense mechanism on the tapping wound of the rubber tree. Regarding substrate specificity and mechanism of action, PPOs are composed of three different types, i.e., tyrosinases, catechol oxidase, and laccases [25]. The enzymatic discoloration is caused by the naturally occurring phenols and amino phenols in NR latex [26]. PPO are the key enzymes in the natural coagulation and darkening of latex [27], and they are located inside the Frey-Wyssling particles. The PPO is responsible for the darkening of coagulated rubber on exposure to air or oxygen, i.e., in the presence of PPO, the phenols and amino phenols combining with oxygen from the air to form *o*-quinones. These *o*-quinones react with naturally occurring amino acids and proteins in the NR latex, giving colored compounds resembling melanin [28,29].

Both PPO and polyphenols are independently present in latex but do not react with each other. However, the Frey-Wyssling particles are destroyed during fractionation and solidification, and enzymes are released into the NR latex serum, leading to discoloration [30,31].

#### 5.1.2. Polyphenol

In the early days, the darkening of rubber was considered a clonal characteristic because RRIC 7 typically produces black rubber. The most common substances associated with enzymatic browning are the cinnamic acids, especially caffeic and chlorogenic acids, tyrosine which is oxidized to a melanin type pigment, and the catechins and leucoanthocyanidins [32]. Many researchers detected tyrosine in NR latex, and at high concentrations, it has often been associated with discoloration of the final rubber [33]. Nadarajah & Karunaratne [29] observed that the phenolic content of RRIC 7 latex was four times that of PB 86 latex. Studies by Yapa [34], Madsaih & Cheewasedtham [35] demonstrated that phenolic oxidation is the main cause of NR blackening.

Among the several phenolic compounds identified, the possible presence of dihydroxyphenylalanine (DOPA) shows a potential pathway for forming dark pigments. DOPA can undergo oxidation, polymerizing to form melanin-type pigments [36].

#### 5.1.3. Effect of Ethephon Stimulation on Polyphenols Content

Stimulation-based low-intensity harvesting (LIH) systems are accepted worldwide as an agronomic tool to overcome the high cost of production, worker scarcity, and low economic lifespan of trees in rubber plantation industries [37]. Discoloration due to ethephon stimulation has been reported [34]; however, there is no evidence for the effect of ethephon concentration on the color of crepe rubber. In 1976, Yapa observed that ethephon could stimulate an increase in the phenol content in the latex exuding from the rubber trees, resulting in slight discoloration of NR [33].

Stimulation by ethephon greatly reduces latex PPO activity, which is correlated positively with plugging index, which results in an increase of phenolic substances in stimulated latex. A significant increase in phenolic content reported (after first & second tapping) after ethephon treatment is due to a decrease in phenol-oxidase activity [35,38]. Brozozowska-Hanower et al. have shown a definite lowering of *o*-diphenol oxidase activity in latices at the first tapping after stimulation of the chaste tree [27].

The ethephon increased the thiol content of the latex, which acted as an antioxidant by reacting with phenolic compounds to form colorless products. It is evident from the results that with increasing ethephon concentration up to 3%, the Lovibond color index is reduced, with the simultaneous increase in thiol concentration. The lighter color observed in rubber with ethephon treatment may prevent phenolic discoloration with thiol compounds. Therefore, thiol concentration above a critical level allows permanent protection against enzymatic browning [39]. However, this level may often be dependent on the physiological condition of the tree [18].

However, there was no significant difference (*p* > 0.05) in Lovibond color among the harvesting systems [40]. Hsia [41] observed that unidentified constituents (whether polyphenols or polyphenol oxides) were rapidly oxidized by a non-enzymatic process immediately after ammoniation of latex. It was concluded that non-rubber constituents were oxidized. The preservation of natural rubber latex with ammonia is still commonly used processing process in the industry. Therefore, the discoloration caused by the appropriate amount of ethephon stimulation is not significant.

### 5.2. Non-Enzymatic Browning

Non-enzymatic browning in natural products has been related to sugar-amino acid condensation [42,43] and ascorbic acid decomposition [44]. There have been few studies of this type in *Hevea* latex. Non-enzymatic browning resulting from the following reactions is possibly concerned with the discolorations of NR. In addition, discoloration possibly involves the oxidation of lipids, especially unsaturated fatty acids [45].

#### 5.2.1. Maillard and Caramelization Reaction

The reaction between sugars and amino groups was first described in 1908 by Ling & Malting, who considered color formation in the beer brewing process. In 1912, Louis-Camille Maillard described a browning reaction between reducing sugars and amino groups. Maillard reaction involves the reaction between carbonyl compounds (reducing sugars, aldehydes, ketones, and lipid oxidation products) and amino compounds (lysine, glycine amine, and ammonia proteins) to produce glycosyl-amino products, followed by Amadori rearrangement. An intermediate step involves dehydration and fragmentation of sugars, amino acid degradation, etc. A final step involves aldol condensation, polymerization, and the formation of colored products.

The condensation products between syrups and amino acids (Amadori compounds) play a role in discoloration in certain types of systems [42,43]. Fructose-tryptophan is among the Amadori compounds, the oxygen-dependent browning of which is one of the most important factors contributing to the browning of soya sauce during storage.

However, little or no attention has been paid to the possible involvement of these Amadori compounds in the darkening processes in NR. Tryptophan was detected significantly in the bottom fraction and *Hevea* latex [46]. Nevertheless, there was not enough evidence to rule out the role of the Amadori compound tryptophan-fructose and several other compounds in the blackening process [34].

That was the case until Montha et al. [47] pointed out that proteins and amino acids in NR latex can react with carbonyl compounds, such as reducing sugars, aldehydes, and ketones, in the presence of heat to produce coloring substances via the Maillard reaction.

Due to banana skin powder being a good source of nutrients, the Maillard reaction occurring in NR latex plays an important role in reducing allergenic protein and increasing the mechanical properties of NR products [48].

**Figure 4:**
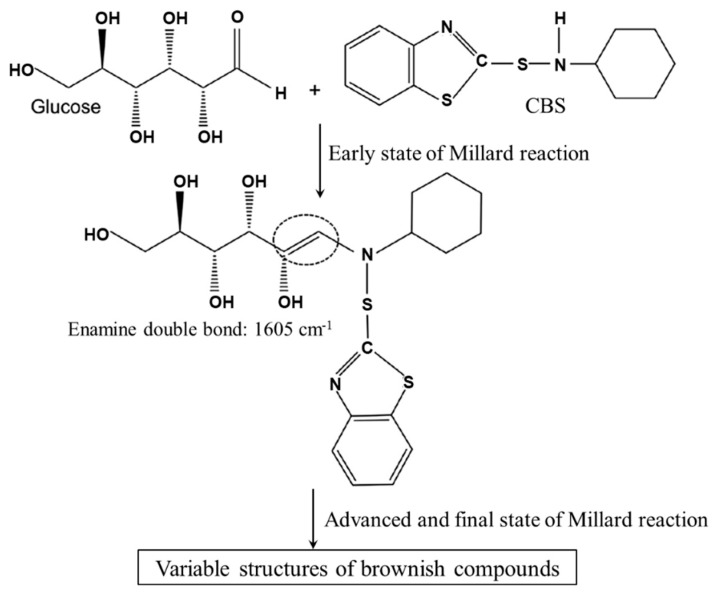
The possible reaction between glucose and CBS produces brownish compounds [49].

Nimpaiboon et al. have shown that the formation of brownish compounds by Maillard reaction occurs in glucose-containing NR after vulcanization due to N-cyclohexyl benzothiazole-2-sulfenamide (CBS), a nitrogenous accelerator [49]. The possible reaction is shown in Figure 4. NR latex contains a small amount of sugar, an important prerequisite for Maillard reaction, leading to rubber discoloration.

Both sulfur and peroxide vulcanizates containing glucose exhibited discoloration and became brown. Moreover, the brown color was darker when the glucose content was increased. Non-enzymatic browning reactions are thought to cause the color change in vulcanizates. Furthermore, the formation of brownish compounds is dependent on the glucose content. In the case of peroxide vulcanization, non-enzymatic browning reactions occur through caramelization only due to the absence of nitrogenous compounds in this system.

On the other hand, both Maillard and caramelization reactions arise after sulfur vulcanization due to CBS, a nitrogenous compound used as an accelerator in this system. Thus, the Maillard reaction can originate from the reaction between glucose and CBS, and it is expected to be the major reaction because the Maillard reaction can occur at a lower temperature than the caramelization reaction. Based on the results in these experiments, it appears that carbohydrates in NR can also cause discoloration, especially in vulcanized products.

#### 5.2.2. Lipid Oxidation

**Figure 5:**
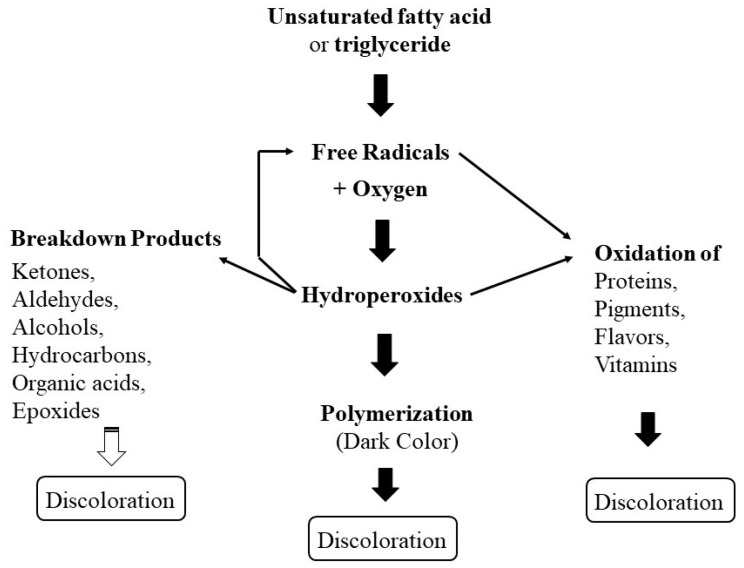
Lipid oxidation mechanisms.

The important lipids involved in oxidation are the unsaturated fatty acid moieties, oleic, linoleic, and linolenic. The oxidation rate of these fatty acids increases with the degree of unsaturation. The mechanism of lipid oxidation is illustrated in Figure 5 [45].

To summarize, discoloration is a prevalent color reaction in NR because of the interaction of phenolic compounds, oxygen, and enzymes, such as PPOs [45]. The browning reaction has two types of reaction in NR. The Enzymatic reaction is when monophonic compounds react with oxygen and PPO, which can produce color polymer or pigments. The other is a non-enzymatic reaction, such as the Maillard reaction, where carbonyl reacts with amino compounds, followed by the dehydration, fragmentation, and aldol condensation reactions which originate the yellow-color product. Furthermore, lipid oxidation also causes the polymerization of unsaturated fatty acids into yellow products, which aggravates the discoloration of NR.

## 6. Methods to Control the Discoloration in Natural Rubber

There is a growing demand for NR products that are lightly colored or not color filled. The principles of browning prevention are essentially the same as those applying to the inhibition of any tissue enzymes, i.e.,;

1. Inhibition or inactivation of the enzyme;
2. Elimination or transformation of the substrate(s);
3. Combination of both above.

In addition, other factors that may cause discoloration need to be considered.

### 6.1. Biologically Controlled Methods

Phenolic compounds could react to form colorless products after enzymatic oxidation with thiols such as cysteine and glutathione [28]. A biologically controlled method can improve the color of crepe rubber with low-frequency harvesting systems with S/2, d3 low-frequency harvesting system, ethephon concentration increased thiol content and a plasticity retention index (PRI) value up to 3% ethephon. Ethephon treated 1–3% samples showed significantly low Lovibond color index (lighter color), and overdosage may lead to discoloration. Lighter-colored rubber is also obtained by adding glutathione at the latex stage to replace the toxic bleach [18]. The first is to obtain high-quality white NR latex from the control source.

### 6.2. Improving the Container

Due to the low price of the plastic container, users do not look with favor on the substitution of a non-metallic container for the steel drum. There is no alternative but to try to apply some protective medium internally. Based on the natural corollary that the discoloration of the latex is due to the formation of amorphous and colloidal iron sulfide in the latex, using zinc instead of iron as the reactive metal should result in a white color, which would make the sulfide invisible to the glasses. Using a latex-zinc oxide film prevents discoloration and minimizes so-called contamination because it stays in the rubber substrate and does not enter the latex [15].

### 6.3. Optimal Centrifugation Process

Rojruthai et al. [50] proposed a more practical and simpler physical process. Process parameters such as % total solid content (TSC) of field latex, centrifugation speed, and duration for centrifugation were optimized to produce the lightest colored concentrated NR latex. Using a batch centrifuge, the optimum conditions for centrifugation of field latex were identified at % TSC less than 35% at 12,000 rpm for 30 min. Washing the latex at least two times during centrifugation will ensure that the lightest colored concentrated NR latex is obtained. The process is feasible for adoption by the industry with the least modification to existing facilities. A light-colored concentrated NR latex can meet the demand for niche NR applications where this additional higher treatment cost is justified.

In addition, Siriwong et al. [51] found that soaking of coagulated NR in hot water (70 °C) for 1 h could significantly reduce the color and the total phenolic content in NR. According to Lovibond colorimeter, the color index of rubber soaking in hot water (W70) is 2.5–3.0, which is much lower than rubber obtained by other processes such as soaked at room temperature (WRT) valued at 4.0–4.5, air dry sheet (ADS) valued at 4.5–5.0, non-soaked NR samples (NoW) valued at 5.0–7.0 and ribbed smoked sheet No.3 (RSS3) valued at 9.0–10.0. It can also be seen that its color is the lightest. Adding a simple hot water soaking process during the preparation of raw rubber can result in a light-colored NR with a high PRI value.

### 6.4. Chemical Treatments

#### 6.4.1. Removal of Non-Rubber Components

One way to reduce the yellow color of NR is to remove non-rubber components, for example, lipids and proteins from NR. The discoloration of NR latex can be prevented by inhibiting either enzymatic activity or eliminating the substrates concerned by chemical treatment or the combination of both [45]. The removal of non-rubber components, in particular proteins and lipids in NR by chemical methods, can help reduce the yellowness index of NR to a level comparable to that of synthetic polyisoprene.

Purifications by deproteinization and transesterification [52] are methods for removing proteins and lipids from NR latex/solution, respectively, that could reduce coloring substances present in NR [53]. Vulcanized NR prepared from highly purified rubber by saponification was light-yellow in color [54,55]. The employment of some chemicals in latex, i.e., a proteolytic enzyme, sodium hydroxide, to reduce rubber discoloration. It was found that the color index of NaOH-treated rubber or saponified rubber (SP) was lightest, whereas the color of urea-treated rubber or deproteinized rubber (DP) was close to the centrifugation rubber. DP and SP may effectively produce light-colored NR [55]. Since non-rubber components were removed into the serum during centrifugation, saponification effectively removes proteins and gives the lightest color film. Thus, these may be effective methods to produce light-colored NR.

#### 6.4.2. Bleaching

Some chemicals are employed to reduce the storage discoloration by inhibiting the enzymatic reactions occurring during rubber storage. Bleaching agents decolorize the naturally occurring pigments present in latex, mostly of the carotenoid type, such as xylyl mercaptan marketed as RPA-3, tolyl mercaptan named PRI-7 [56].

Adding the appropriate amount of bleach is key to treatment, i.e., if the excess is added, the rubber tends to soften, especially when exposed to sunlight or heat. The addition of lower quantities than the recommended dosage will produce crepe rubber which may go into the lower grades. Therefore, the strength or the active ingredient content of these bleaching agents should be determined [56]. Another method for controlling discoloration was revealed by adding thiols such as cysteine and glutathione at the latex stage to replace the toxic bleaching agents [28], thereby lightening the color of the rubber.

#### 6.4.3. Addition of Strong Reducing Agent

NR latex discoloration can also be prevented by inhibiting either enzymatic activity or eliminating the substrates concerned by chemical treatment, or combining both [45]. Sodium metabisulphite (SMS) is a stronger reducing agent than PPO, an enzyme accelerator for forming color substances in NR. Thus, SMS can inhibit the formation of color substances in NR. In China, the production of standard light-colored rubber usually uses SMS as a reducing agent. However, it has been confirmed that adding SMS only slightly reduced the color of the rubber and showed an insignificant effect on the decoloring [55].

Discoloration of crepe rubber is normally inhibited or prevented by using chemicals, e.g., oxalic acid, to retard enzymatic discoloration [57] and sulfites for non-enzymic browning [58]. Skim rubber is a by-product of field latex through centrifugal production of concentrated NR latex, and the color is black and brown after sulfuric acid treatment and solidification. Oxalic acid can be used to bleach rubber and make the color of the rubber lighter [59]. The initial plasticity value (P_0_), plasticity retention index (PRI), mechanical properties, and aging properties were improved when the optimum amount of oxalic acid was 2%. Oxalate has a strong reductive property, which can prevent the oxidation and discoloration of PPO and chelate with copper ion, which affects the aging resistance, thus improving the aging resistance of skim rubber. Hence, the clarity of the final product is achieved mainly by fractionation and/or bleaching in the production of crepe rubber from NR latex.

### 6.5. Advanced Drying Technology

Due to its low conductivity and dielectric properties, the rubber drying process requires intensive thermal energy and an extended drying period. Drying rubber at high temperatures and/or for an extended period could accelerate the coloration of NR, turning it a yellowish or brown color [60]. Thus, selecting advanced drying techniques of crumbs rubber is an important measure to improve drying efficiency and reduce discoloration.

Unlike other conventional drying methods, microwave heating does not rely on the conduction of heat through solids such as rubber but rather through self-excited molecules, particularly for polar materials due to absorption of microwave energy. Microwave drying on grape and carrot samples indicated that microwave drying was better than hot air drying in retaining product color. However, the microwave heating applied in crumb rubber drying is not cost-effective and as yet too capital intensive from the economic point of view [61]. Microwave drying shall be associated with other techniques such as forced air or vacuum to complete the drying and further enhance the process efficiency.

## 7. Conclusions

Color is a very important aspect of natural rubber and its products, which determines the technical classification, economic value, and applicability of NR. The dark color developed in dry rubber is a major issue in some industries which require a pale color raw material. We reviewed the development history of light-colored NR, the origin and species of NR color, and the discoloration mechanism and provide strategies for developing light-colored rubber.

According to the enzyme darkening phenomenon and the pigment bleaching technology principle, referring to the modern processing technology of light-colored rubber, we put forward the suggested strategy for the production of high-quality light-colored standard rubber: with the selection of high-quality field rubber latex (clones such as PB86, RRIM 600) as raw material, making good early preservation of NR latex before processing, by strictly following the standardized procedures and advanced technology for production, supplemented by an appropriate amount of reducing agent to reduce enzymatic and non-enzymatic browning, the pigment-bleaching method and soaking of coagulated NR in hot water functions as a combined technical route.

## Data Availability

No new data were created or analyzed in this study. Data sharing is not applicable to this article.
